# Correlation of Histopathology and Clinical Symptoms in Meralgia Paresthetica

**DOI:** 10.7759/cureus.1789

**Published:** 2017-10-20

**Authors:** Russell A Payne, Kimberly Harbaugh, Charles S Specht, Elias Rizk

**Affiliations:** 1 Department of Neurosurgery, Penn State Hershey Medical Center; 2 Pathology, Penn State Hershey Medical Center

**Keywords:** meralgia paresthetica, nerve entrapment, lateral femoral cutaneous nerve

## Abstract

Meralgia paresthetica is a neuropathic pain disorder resulting from an entrapment neuropathy of the lateral femoral cutaneous nerve. This condition results in pain, paresthesias and numbness over the anterolateral aspect of the thigh. We present a case of meralgia paresthetica and discuss both the clinical and histopathological findings as they relate to one another. We report a case of meralgia paresthetica refractory to conservative treatment who underwent neurectomy with successful treatment of symptoms. Histopathological examination revealed moderate loss of myelinated axons with some axonal atrophy. The distinct pathologic findings were axonal regeneration clusters and thinly myelinated axons as well as moderate perineurial thickening. These findings corresponded well to the patient’s preoperative symptoms of paresthesias and pain. This case serves to shed light on the pathophysiology of meralgia paresthetica and its clinical presentation. It also shows the role of surgical treatment in cases refractory to conservative management in order to alleviate painful symptoms

## Introduction

Meralgia paresthetica (MP) is a neuropathic pain disorder with an intriguing history. Its name is derived from the Greek word meros (thigh) and algos (pain) and was first described in the late 19th century by Martin Bernhardt, a German pathologist [1-2]. MP was later described by Vladimir Karlovich Roth in cavalry soldiers with tight belts and garnered the eponym Bernhardt-Roth syndrome. Perhaps most interesting is that Sigmund Freud suffered from this condition and wrote about its unrelenting and agonizing symptoms in a German medical journal [3]. MP is an entrapment neuropathy of the lateral femoral cutaneous nerve (LFCN) that results in pain, paresthesias, and numbness over the anterolateral aspect of the thigh. MP usually occurs in decades four and five. It show s a predilection for males, and commonly afflicts individuals who are obese, diabetic or pregnant [4]. Patients typically describe a dysesthetic burning or tingling over the LFCN distribution (anterolateral thigh). Physical examination reveals tenderness where the LFCN and inguinal ligament meet with worsened pain when the ligament is tapped or when the leg is extended. Diagnosis chiefly relies on clinical history and physical examination; though, imaging and electrodiagnostic studies may be useful adjuncts [5]. Histopathological evaluation reveals findings consistent with a LFCN mononeuropathy with reduced myelinated nerve fiber density, perineurial thickening, subperineurial edema and occasional regenerating nerve clusters [6].

## Case presentation

We report a case of a patient with MP refractory to conservative treatment and lateral femoral cutaneous neuropathy (LCFN) neurolysis who underwent neurectomy for definitive treatment. The sectioned nerve was sent for histopathological analysis with the goal of correlating histopathological findings with MP symptomatology.

The patient is an obese 23 year-old woman, with a body mass index (BMI) of 43, who developed dysesthetic anterolateral left thigh pain and was diagnosed with LFCN entrapment. She underwent neurolysis of the nerve without improvement. A unilateral postoperative nerve conduction study (NCS) showed no response from the left LFCN with normal latencies and amplitudes seen in the sural, peroneal and tibial nerves. A needle electromyography (EMG) of the left extremity evaluated the gluteus medius, vastus lateralis, vastus medialis, tibialis anterior, medial gastrocnemius and medial hamstrings muscles. These showed normal membrane stability, normal motor unit action potential morphology, and normal recruitment. A pelvic magnetic resonance imaging (MRI) showed no abnormalities. She underwent an ultrasound-guided LCFN injection with 1% lidocaine resulting in temporary relief. Examination revealed decreased sensation in the LCFN nerve distribution as well as tenderness in the region of the anterior superior iliac spine (ASIS). Palpation of this area reproduced the pain in the LFCN distribution. She then elected to undergo surgery with transection of the LFCN and was taken to the operating room where a portion of the nerve was sectioned and sent to pathology. At her three month post-operative follow-up, she showed improvement with reduction of pain and discomfort over the LFCN distribution. She suffered no new deficits from the surgery but did continue to have decreased sensation in the LCFN distribution.

**Figure 1:**
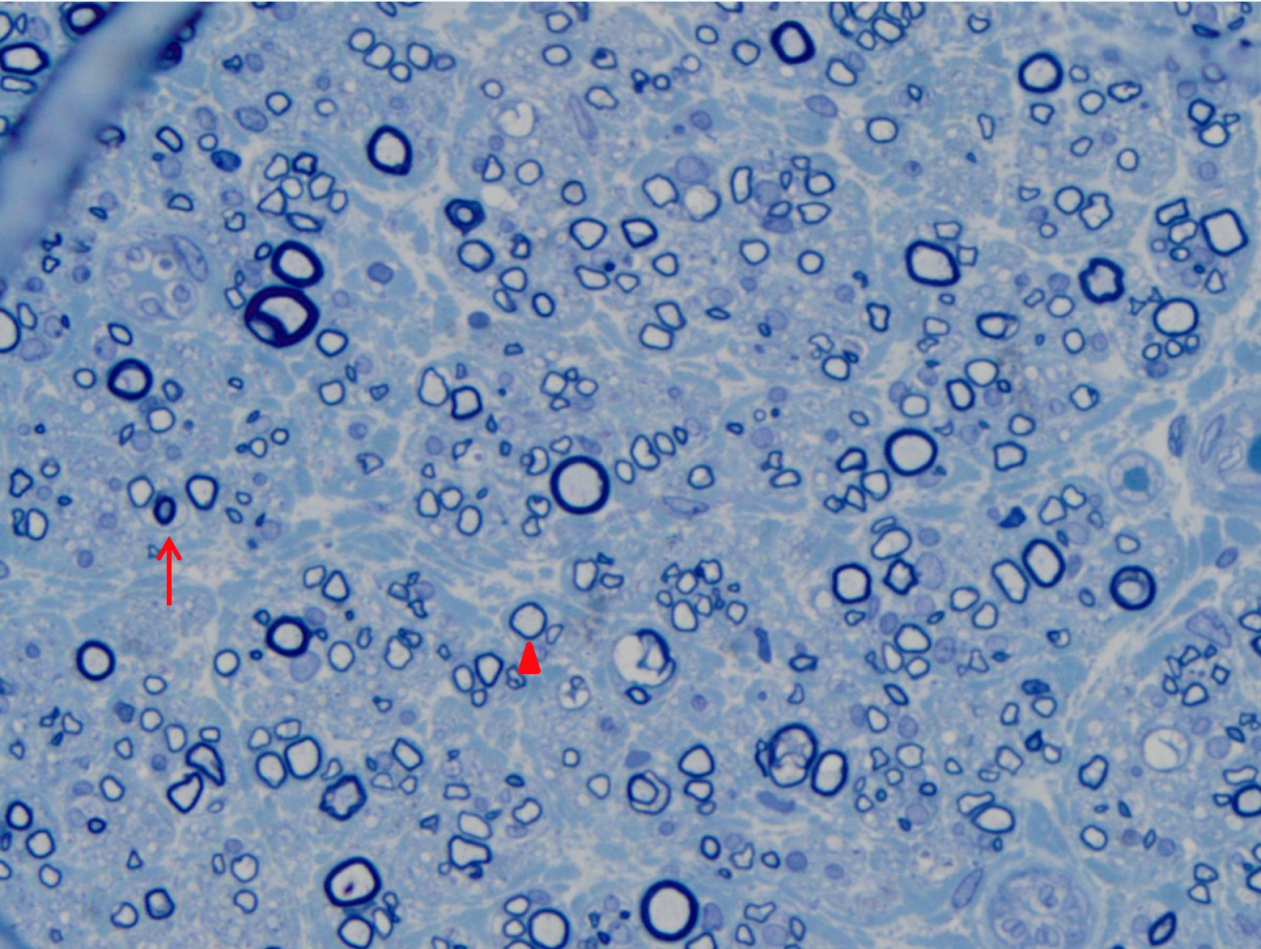
Toluidine blue, 1200x Thinly myelinated axons consistent with demyelination (arrowhead). A profile of axonal atrophy can also be seen (red arrow).

Gross examination revealed a 1.8 cm segment of peripheral nerve tissue (LFCN). Toluidine blue-stained resin sections showed moderate loss of myelinated axons with some axonal atrophy (Figure 1).

**Figure 2:**
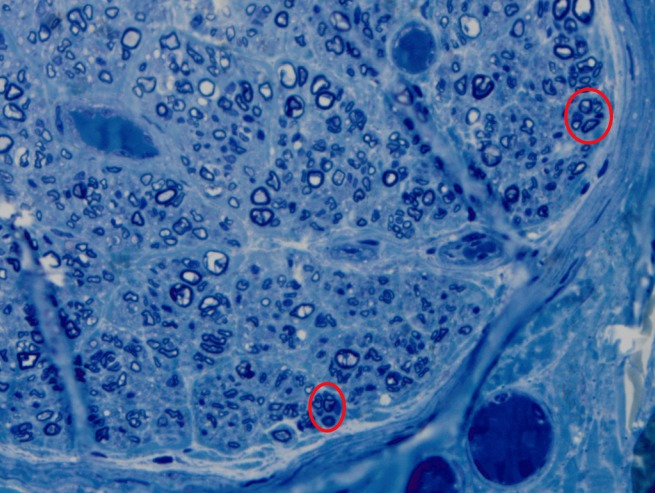
Toluidine blue, 1200x Regenerative nerve clusters shown by small clustered nerve fibers (red circles).

Axonal regeneration clusters and thinly myelinated axons were scattered throughout the specimen indicating some measure of axonal regrowth and recovery (Figure 2). 

**Figure 3:**
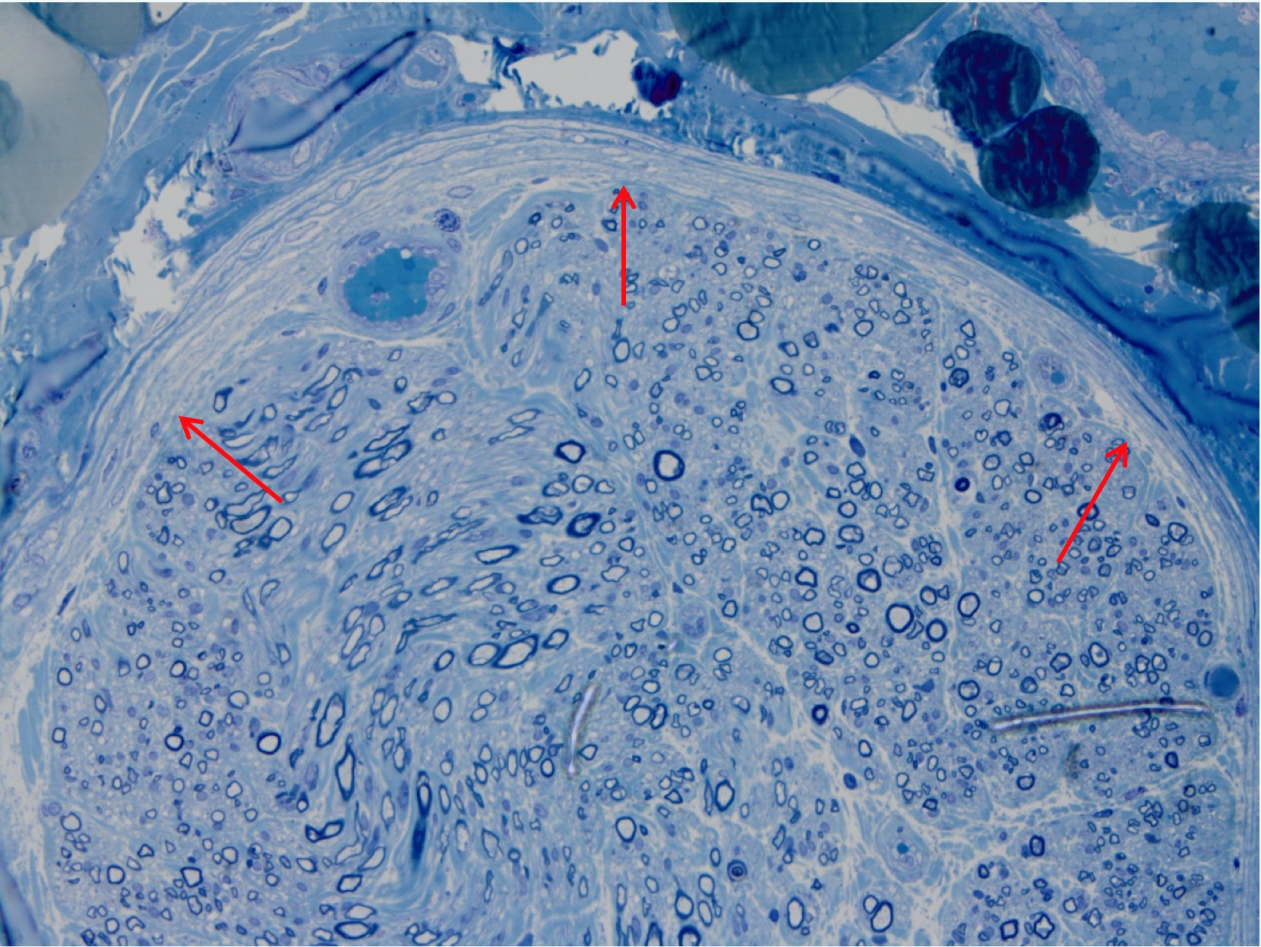
Toluidine blue, 400x Thickened perineurium surrounding the nerve tissue (red arrows).

Moderate perineurial thickening was present again indicating an ongoing injurious process (Figure 3).

## Discussion

Meralgia paresthetica is a well-known cause of anterolateral thigh pain and results from entrapment of the LFCN. Our unique findings of axonal loss and demyelination with scattered regeneration clusters are consistent with a compressive neuropathy, similar to findings in carpal tunnel syndrome [7]. Compression of the LCFN fibers under the attachment of the inguinal ligament to the ASIS is thought to cause perineurial edema and fibrosis which progresses to focal and then diffuse demyelination and finally culminates in axonal loss and inflammation [6]. Scattered regeneration clusters are seen when axonal loss results in changes in genetic expression which then causes an increase in neurotrophic factors and their receptors to support axon elongation from the nerve stump [8].

Demyelination and axonal loss of LCFN fibers explain the dysesthesias, numbness, and pain experienced by patients and the lack of signals on electrodiagnostic evaluation. The thickening of the perineurium and the presence of scattered regeneration clusters indicates an ongoing injurious process. These findings are consistent with a compressive neuropathy and suggest that in order to adequately treat the symptoms of chronic idiopathic MP, the site of compression can be surgically addressed when conservative measures fail, options include neurolysis and neurectomy. Some have suggested that the area of pathology in the LCFN showing demyelination, axonal loss and perineurial thickening is a pain generator and have therefore advocated for neurectomy [6,9]. Others have suggested that it should be treated in the same manner of other compression neuropathies with neurolysis. The optimal surgical treatment for MP is debated in the literature and cannot be determined due to the low quality of evidence [10].

## Conclusions

We present a patient with MP confirmed by electrodiagnostic testing who failed conservative treatment. Surgery resulted in improvement in symptoms. Findings within the sectioned nerve showed axonal loss, demyelination and perineurial thickening which explained the symptoms of painful numbness experienced by this patient. This case shows the correlation between histopathological findings and symptoms in MP and highlights the importance of surgical treatment of chronic idiopathic MP with neurectomy or neurolysis when conservative treatment has failed.
